# Classification of pain intensity with the pain beliefs and perceptions inventory (PBPI) and the pain catastrophizing scales (PCS)

**DOI:** 10.1007/s11136-023-03444-8

**Published:** 2023-05-26

**Authors:** Angel Blanch, Sílvia Solé

**Affiliations:** 1 Department of Psychology, University of Lleida, Lleida, Spain; 2 Department of Nursing and Physiotherapy, University of Lleida, Lleida, Spain; 3 Department of Physiotherapy, Andalucia TECH, University of Malaga, Málaga, Spain

**Keywords:** Pain beliefs, Pain catastrophizing, Pain intensity, ROC curves

## Abstract

**Purpose:**

The pain beliefs and perceptions inventory (PBPI) and the pain catastrophizing scales (PCS) characterize beliefs or distress dimensions of the pain experience. It is relatively unknown, however, to what degree the PBPI and the PCS are well suited to classifying pain intensity.

**Methods:**

This study applied a receiver operating characteristic (ROC) approach to these instruments against the criterion of a visual analogue scale (VAS) of pain intensity with fibromyalgia and chronic back pain patients (*n* = 419).

**Results:**

The largest areas under the curve (AUC) were moderate and limited to the constancy subscale (71%) and total score (70%) of the PBPI and to the helplessness subscale (75%) and total score (72%) of the PCS. The best cut-off scores for the PBPI and PCS were better off at detecting true negatives than true positives, with larger specificity than sensitivity values.

**Conclusion:**

Whereas, the PBPI and PCS are certainly useful instruments to evaluate diverse pain experiences, they may be inappropriate to classify intensity. The PCS performs marginally better than the PBPI for classifying pain intensity.

**Supplementary Information:**

The online version contains supplementary material available at 10.1007/s11136-023-03444-8.

Several psychometric instruments are routinely used to assess the experience and the endurance of patients with chronic pain. Two well-known instruments in this field of research are the Pain Beliefs and Perceptions Inventory (PBPI) and the Pain Catastrophizing Scale (PCS), which have been extensively used in both basic and applied research [1–3].

The PBPI evaluates pain-related perceptions, affect, and behaviour. There are robust associations between this instrument with pain quality, depression, anxiety, personality traits, physical functioning, and coping [4]. The PCS also evaluates beliefs about pain focussing on the pain experience as unmanageable and stressful. Catastrophizers tend to report more negative thoughts about pain and higher emotional distress compared with non-catastrophizers, including more pain intensity when exposed to an ice water immersion procedure [5]. The PCS correlates negatively with positive emotions, such as happiness, love, or pride, and positively with negative emotions, such as sadness, hate, or fear. Moreover, the PCS differentiates well between patients and community individuals or between males and females [6].

The PBPI comprises four factors, permanence, constancy, mystery, and self-blame. The PCS comprises three factors, rumination, magnification, and helplessness. The factor structures of the PBPI and PCS are generally sound and replicable across different languages, such as Chinese [7, 8], Spanish [9], German [10, 11], Italian [12, 13], or Portuguese [14, 15]. Furthermore, both measures bear sound psychometric properties and their use is widespread in basic and applied research settings.

The PBPI and the PCS may be useful to select patients suffering from chronic pain to undergo cognitive-behavioural therapy targeting pain management, because patient’s beliefs about pain modulate behaviour, physical condition, and response to treatment. For example, patients experiencing back pain are more prone to anxiety and depressive disorders, which might impair successful therapeutic interventions [16]. Furthermore, there are more robust links between the PBI and the PCS with pain intensity than with other self-reported psychometric measures [4, 5]. These relationships, however, may vary depending on specific instrument subscales. For instance, there is a robust correlation between pain intensity and the PBPI constancy scale [14] and a moderate correlation between the PCS and the perception of pain severity in a nonclinical population [6]. Moreover, scoring high in the PCS correspond to higher cut-off points on a numerical rating scale (NRS) of pain intensity [17]. Similarly, pain intensity might be reduced with clinical interventions focussed on catastrophizing in patients with acute back pain [1]. In the light of these findings, some researchers might consider the PBPI and PCS as appropriate on some instances to evaluate the magnitude of pain intensity.

Whether these scales may be capable to differentiate between high or low levels of pain intensity, however, remains relatively unknown. The current study intends to shed light on this issue, by comparing the scales and whole test scores of the PBPI and PCS against a visual analogue scale (VAS) measuring pain intensity. The study main aim was to evaluate the classification properties of the PBPI and the PCS regarding pain intensity outcomes derived from the VAS. A secondary aim was to provide the cut-off values of the PBPI and PCS subscales yielding the best sensitivity, specificity, accuracy, and precision values. A receiver operating characteristic curve procedure (ROC) was used to address these aims.

## Method

### Participants

After obtaining the ethics committee approval, patients were recruited from the fibromyalgia unit of a hospital and two primary care centres in North-eastern Spain between February 2020 and March 2021. Patients were invited to participate in this study during their regular visits to a medical centre where a project about pain management was implemented. There were 376 patients (90%) diagnosed with fibromyalgia and 43 patients (10%) diagnosed with chronic back pain (*n* = 419).

This was a retrospective study that was not preregistered. The main inclusion criterion was a medical diagnosis of fibromyalgia or chronic low back pain. Additional inclusion criteria included an age of 18 years old or above and fluency in Spanish language. The exclusion criteria were central or peripheral neurological signs, mental impairment, and no signing of the corresponding informed consent. A research assistant through online surveys collected the pain and sociodemographic characteristics during COVID-19 lockdown and restrictions.

### Measures

The PBPI is a questionnaire with 16 items that comprises four main dimensions of pain beliefs, mystery, permanence, constancy, and self-blame [18]. Items are scored with a 4-point scale, from -2 (*fully disagree*) to 2 (*fully agree*). Four items are reverse scored: 3 (constancy), 9, 12, and 15 (permanence). The permanence dimension evaluates whether it is believed that pain is part of life. The constancy dimension evaluates the belief in the endurance of pain throughout daily life. The mystery dimension refers to the mystery and understanding of pain. The self-blame dimension evaluates whether it is believed that one is the sole responsible of experiencing pain.

The PCS is a 13-item questionnaire comprising three factors, rumination, magnification, and helplessness [5]. Items are scored with a 5-point scale from 0 (*not at all*) to 4 (*all the time*). The rumination factor taps ruminative thinking, worry, and exacerbation of pain-related thoughts. The magnification factor encompasses the exaggeration of the painful experience and focus on negative outcomes. The helplessness factor implies the incapacity to cope with pain.

A visual analogue scale (VAS) for pain intensity was used as the criterion variable in this study. The use of a VAS for assessing pain has been deemed as a proper, easy, and direct device to assess the affective magnitude of chronic and experimental pain [19]. This scale consisted in a single item that was scored from zero (no pain) to 10 points (maximum pain). This item was displayed as a 10 cm (cm) horizontal line. Patients were asked to draw a vertical mark in the line point thought to represent their current perception of pain. The item was scored by measuring the distance from the zero point to the participant’s mark, rounded off to the next integer such as 4.4 was rounded off to 4, whereas 6.5 was rounded off to 7.

**Fig. 1:**
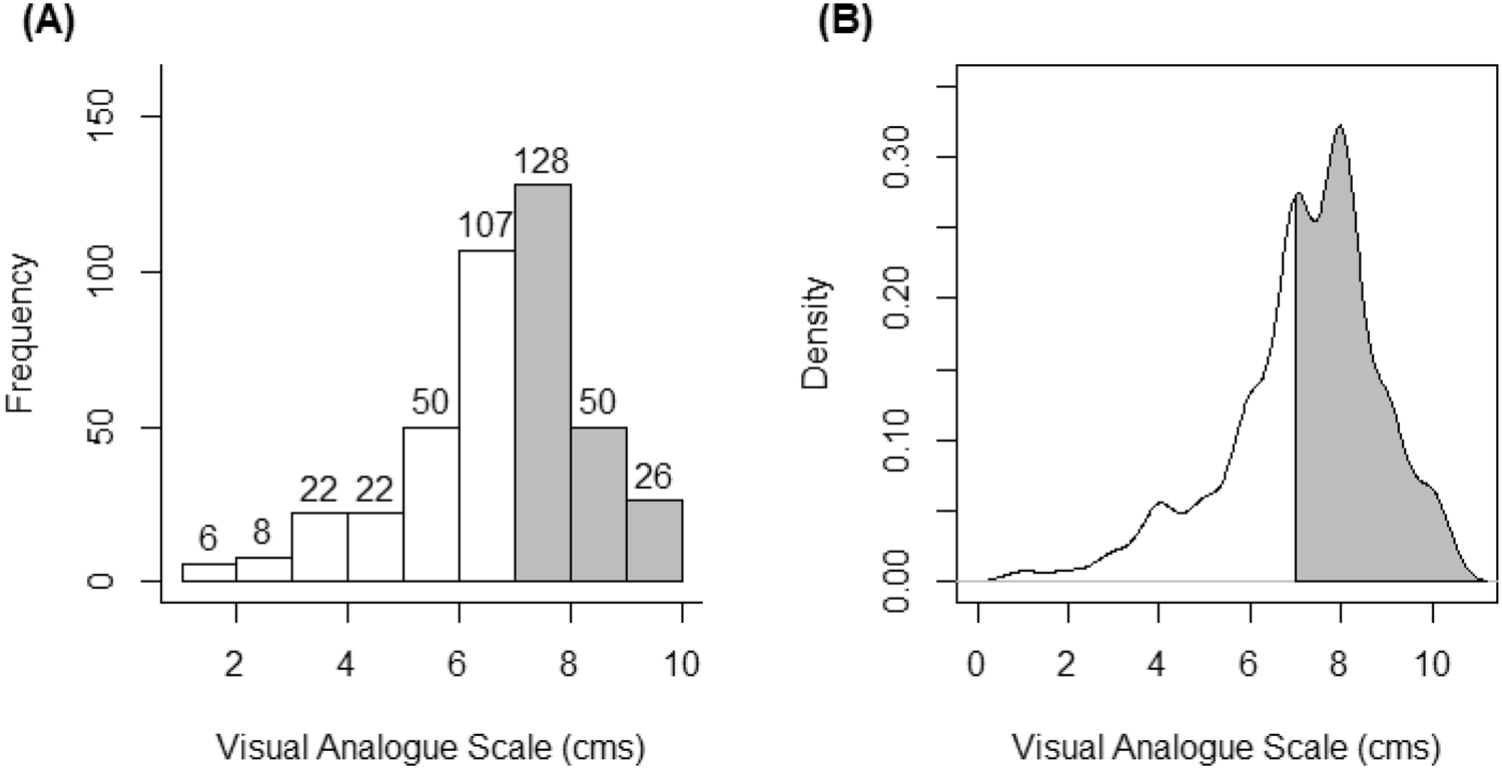
Frequency distribution (**A**) and probability density function (**B**) of the Visual Analogue Scale (VAS). White bars and areas are low pain (*n* = 215) and grey bars and areas are high pain (*n* = 204) at a VAS cut-off point of 7

Past research with the VAS has determined cut-off values of this scale for mild, moderate, and severe pain intensity, assuming the VAS as the independent variable and being contrasted with the Short Form Health Survey (SF-36) and the verbal rating scale (VRS) of pain intensity as dependent variables [20]. In the current study, the VAS was used as the dependent variable or classification criterion regarding the diagnostic performance of the PBPI and the PCS. For the current study with fibromyalgia and chronic back pain patients, two groups with a very similar number of participants were determined from the median score to the VAS (median = 7 points) as shown in Fig. 1. Scores equal or below 7 were considered as low pain (*n* = 215, 51% of the total sample) and scores above 7 were considered as high pain (*n* = 204, 49% of the total sample).

### Data analyses

A descriptive analysis was conducted to evaluate the differences in the PBPI and PCS subscales between fibromyalgia and chronic back pain patients (*d* effect sizes), the Cronbach’s alpha reliability of the PBPI and PCS subscales, and the Pearson correlation of the PBPI and the PCS subscales with the VAS.

Receiver operating characteristic (ROC) curves were estimated for the total scores and for each of the PBPI and PCS subscales. This is an appropriate method to evaluate and compare the diagnostic and classification capabilities of two or more psychometric tests [21].

We first evaluated whether the target psychometric tests were able to discriminate between low pain (*n* = 215) and high pain (*n* = 204) in accordance with the VAS. The ability to discriminate between both groups was evaluated by estimating the area under the curve (AUC). In general, the discriminative ability is low when AUCs are between 0.5 and 0.7, moderate between 0.7 and 0.9, and high when above 0.9. In the second place, we determined the best cut-off scores of each test that yielded the higher sensitivity, specificity, accuracy, and precision. For comparative purposes, these scores were provided in standardized form (*Z*) with a mean of zero (*Sd* = 1).

The ROC curves and best cut-off scores were estimated with the R software and the pROC package [22, 23]. Materials and analysis code for this study are available by emailing the corresponding author.

## Results

Fibromyalgia patients scored higher than patients with chronic back pain in some PBPI and PCS scales, with rather modest effect sizes in the comparison of both groups of patients [24, 25]. In the PBPI effect sizes (*d*, [95% CI]) were low for Self-blame (*d* = 0, [− 0.31, 0.32]) and Mystery (*d* = 0.14, [− 0.18, 0.45]), medium for Permanence (*d* = 0.52, [0.21, 0.84]) and for the total PBPI score (*d* = 0.59, [0.27, 0.91]), and large for Constancy (*d* = 0.91, [0.59, 1.23]). Moreover, effect sizes were low for Rumination (*d* = 0.37, [0.05, 0.68]) and Magnification (*d* = 0.41, [0.10, 0.73]) and medium for Helplessness (*d* = 0.62, [0.30, 0.94]) and the total PCS score (*d* = 0.51, [0.19, 0.82]). The effect size for the VAS was large (*d* = 0.82, [0.5, 1.14]). The ROC outcomes described next for all participants did not differ meaningfully from the ROC outcomes conducted with the fibromyalgia patients only.

Table 1 shows the number of items and the Cronbach’s alpha reliabilities for the PBPI and the PCS, together with their Pearson correlations with the VAS. Most of the subscales and total score in both tests, PBPI and PCS, had acceptable to very good Cronbach’s alpha reliabilities, with values equal or above 0.70. Only the Cronbach’s alpha reliability in the Permanence PBPI subscale was questionable with a value of 0.67. The Pearson correlations with the VAS were somewhat lower for the PBPI than for the PCS, ranging from 0.12 (Self-blame) to 0.45 (Constancy) for the PBPI and from 0.36 (Magnification) to 0.48 (Helplessness) for the PCS.

**Table 1:** Number of items, Cronbach’s alpha reliability, and Pearson correlation coefficients of the Pain Beliefs and Perceptions Inventory (PBPI) and the Pain Catastrophizing Scale (PCS) with the Visual Analogue Scale (VAS)

Scale	Number of items	Reliability (α)	Pearson correlation with the visual analogue scale (VAS)	*p*-value
Permanence	5	.67	.29	8.26e-10
Constancy	4	.74	.45	2.2e-16
Mystery	4	.76	.19	.0001
Self-blame	3	.70	.12	.0124
PBPI	16	.80	.39	2.2e-16
Rumination	4	.93	.41	2.2e-16
Magnification	3	.84	.36	6.366e-14
Helplessness	6	.93	.48	2.2e-16
PCS	13	.96	.44	2.2e-16

**Fig. 2:**
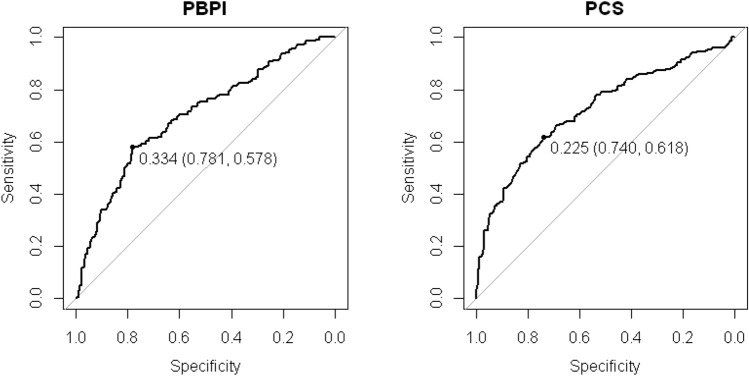
ROC curves for the total scores of the PBPI and PCS with the best cut-off scores (Specificity, Sensitivity)

Table 2 shows the best cut-off values (*Z*) that yielded the higher sensitivity (SEN) and specificity (SPE) of the PBPI and PCS scales when classifying low pain and cases in the VAS. True positives and negatives (TP, TN) and false positives and negatives (FP, FN) are shown in the Appendix. These best cut-off scores failed to provide a good discrimination of low pain and high pain in the evaluation of pain intensity with the VAS. In all subscales, the sensitivity values were below 70%. The specificity values were slightly higher, even though below an optimal discriminative ability of 90%. Table 2 also shows that the areas under the curve (AUCs) ranged between 0.561 for the Self-blame subscale of the PBPI and 0.746 for the Helplessness subscale of the PCS. The AUCs for the full PBPI and PCS were 0.701 and 0.722, respectively. These figures indicate that the discriminative ability of these scales between the two groups determined by the VAS (low pain and high pain) was in the low to moderate range (See Fig. 2).

**Table 2:** Best cut-off values (*Z*), sensitivity (SEN), specificity (SPE), accuracy (ACC), precision (PRE) of the Pain Beliefs and Perceptions Inventory (PBPI), and the Pain Catastrophizing Scale (PCS). Area under the curve (AUC) with 95% confidence interval (CI)

Scale	*Z*	SEN	SPE	PRE	ACC	AUC	95% CI
Permanence	.062	.608	.614	.599	.611	.637	.585, .690
Constancy	.342	.618	.721	.677	.671	.709	.660, .758
Mystery	.340	.431	.730	.603	.585	.604	.551, .658
Self-blame	.949	.201	.898	.651	.559	.561	.507, .614
PBPI	.334	.578	.781	.715	.683	.701	.651, .751
Rumination	.137	.667	.684	.667	.675	.709	.659, .759
Magnification	.324	.539	.754	.675	.649	.672	.620, .723
Helplessness	.506	.539	.842	.764	.695	.746	.699, .793
PCS	.225	.618	.740	.692	.680	.722	.673, .771

With the sensitivity measure tapping the probability of a positive result with a present disorder and the specificity measure tapping the probability of a negative result with an absent disorder, it appears that the PBPI and PCS scales were slightly better off at detecting the absence rather than the presence of high pain intensity. Precision (PRE) values, however, were also below 70% highlighting a rather low probability of pain intensity as currently evaluated with the VAS and the PBPI and PCS best cut-off scores. Moreover, the low accuracy (ACC) values ranging between 0.559 (PBPI, Self-blame) and 0.695 (PCS, Helplessness) reflected a poor clinical performance of the PBPI and PCS to detecting pain intensity.

## Discussion

The PBPI and the PCS were designed to tap constructs associated with the overall experience of pain, which have been also suggested to associate with the magnitude of pain intensity [4, 5]. The current study was two folded. The primary aim was to evaluate whether the PBPI and the PCS classified well the pain intensity of patients measured with a visual analogue scale (VAS). Moreover, this study provides the best cut-off values of the PBPI and PCS that correctly classified fibromyalgia and chronic back pain patients participating in the current study.

There was a low discriminative ability of both instruments to distinguishing between low and high scorers in pain intensity, with areas under the curve of around 70% [21]. The areas under the curve for the PBPI subscales ranged between 56 and 71% and for the PCS subscales ranged between 67 and 75%. These findings suggest that the PBPI and PCS scales were unable to classify patients according with a pain intensity measure, such as the VAS. The PCS, however, appeared slightly better than the PBPI at distinguishing groups of patients with either low or high pain intensity. This outcome aligns somehow with the positive correlations between catastrophizing and higher pain intensity reported with the current data and elsewhere [5, 17].

The PBPI evaluates patients’ thoughts and beliefs about their own pain and the ways of coping with pain, including pain intensity [4]. The present outcomes indicated a lower number of true positives but a larger number of false negatives in the Mystery and Self-blame subscales compared with the Permanence and Constancy subscales (see Online Appendix). Moreover, there were indeed weaker Pearson correlations (Table 1) between the VAS pain intensity measure with the Mystery and Self-blame subscales (0.19 and 0.12) compared with the Permanence and Constancy subscales (0.29 and 0.45). These outcomes could be explained because Mystery and Self-blame evaluate more subjective experiences concerned with the individual understanding and responsibility of pain, which might bear weaker links to the actual experience of pain intensity assessed with the VAS. On the other hand, the Permanence and Constancy PBPI subscales focus on the daily life experiences of pain, which might bear a stronger link to the actual experience of pain intensity assessed with the VAS.

The PCS is considered one of the most important instruments to evaluate the psychosocial experiences of patients enduring chronic pain [5]. In contrast with the PBPI, the PCS subscales (Rumination, Magnification, and Helplessness) yielded a slightly better balance between true positives/negatives with false positive/negatives than the PBPI Mystery and Self-blame subscales. Furthermore, the PCS showed more robust Pearson correlations with pain intensity, suggesting that patients scoring higher in the PCS endured a higher level of pain intensity, as highlighted in earlier findings with different measures of pain intensity [6, 17]. The present outcomes suggest a somewhat larger classification capacity of the PCS than the PBPI regarding low pain and high pain determined with the VAS. Areas under the curve (AUCs) were very close or above the 70% for all subscales of the PCS, whilst for the PBPI there were only three out of four subscales with AUCs around the 60%. In the light of the current findings, however, neither the PBPI nor the PCS appear as usable to distinguish between different levels of pain intensity.

This differential classification ability between the PBPI and the PCS might partly stem in the nature of the fibromyalgia disorder endured by most participants in the current study. Fibromyalgia is a complex disease affecting about 2% of the general population, involving chronic widespread musculoskeletal pain and fatigue, to major health problems, such as sleep disorders, fear, phobias, anxiety, and depression [26]. Fibromyalgia patients withstand persistent cognitive biases and maladaptive thoughts, enhancing the experience of higher levels of pain intensity [26]. The evaluation and treatment of fibromyalgia require a multi-component biopsychosocial approach combining pharmacological and non-pharmacological treatments. For example, pain education protocols addressing chronic pain management focus on pain perception and beliefs for the prognosis and evolution of fibromyalgia [27]. Therefore, the use of specific scales for the evaluation of psychosocial factors related with pain becomes especially important, including the PBPI and PCS. Nonetheless, and according with the outcomes in the present study, the PBPI or the PCS is inappropriate to evaluate the magnitude of pain intensity even with fibromyalgia patients.

A limitation in this study was that the group was slightly heterogeneous regarding the disorder involved with pain. The 10% of the group of participants (43 patients) were diagnosed with chronic back pain, who scored lower than fibromyalgia patients in a few scales, including the VAS. Removing the chronic back pain patients, however, yielded analogue outcomes as those reported so far. In any case, this point should be considered in further replication studies about this topic. The current study outcomes were constrained to patients diagnosed with fibromyalgia or chronic back pain. Future research in this field could replicate whether the PBPI and PCS are able to classify high pain and low pain in pain intensity with other disorders involving the deleterious influence of pain.

Furthermore, this study used as a criterion a single-item visual analogue scale (VAS) tapping current pain intensity, which may have affected the reported classification outcomes. Alternative answering schemes, including pain at the moment, average last week, worst last week, best last week, or average last month, could imply different classification outcomes [20]. For instance, the PBPI and the PCS might be more influenced by worst pain intensity than by current pain intensity evaluated in this study, which might perhaps be a more stable and consistent measure of pain intensity. Relatedly, the cut-off point used here was the median in the VAS to build two groups with an equivalent number of individuals, which was a rather high score of 7 in a scale from zero to 10. It could be argued, therefore, that the conclusions derived from the study might only be valid for populations of patients with analogue cut-off points. Applying different cut-off points to single-item scales such as the numeric rating scale (NRS) or the visual analogue scale (VAS) provides varying outcomes regarding pain experiences evaluated with psychometric instruments as reported elsewhere [17, 20].

The evaluation of pain experiences in clinical settings is regularly conducted with psychometric instruments such as the pain beliefs and perceptions inventory (PBPI) or the pain catastrophizing scales (PCS). These instruments, however, appear rather inappropriate for classification purposes of pain intensity, although the PCS appears as slightly more promising than the PBPI.

## Supplementary Information

Below is the link to the electronic supplementary material.

Supplementary file1 (DOCX 17 kb)

## Data Availability

The data and code are available from Sílvia Solé (silvia.sole@udl.cat) on request.
