# Supplementary material for: Classification of pain intensity with the pain beliefs and perceptions inventory (PBPI) and the pain catastrophizing scales (PCS)
Source: Qual Life Res. 2023 May 26;32(10):2853–9. doi: 10.1007/s11136-023-03444-8 (PMC10214345; doi:10.1007/s11136-023-03444-8)
Supplement: Supplementary file 1 — Supplementary file1 (DOCX 17 kb) [file 11136_2023_3444_MOESM1_ESM.docx]

Appendix. True positives and negatives (TP, TN) and false positives and negatives (FP, FN) in the PBPI and PCS subscales when classifying participants into the high pain (*n* = 204) and low pain (*n* = 215) groups in the Visual Analogue Scale (VAS) at a cut-off value of 7 points.

| Scale |  | TP | TN | FP | FN |
| --- | --- | --- | --- | --- | --- |
| Permanence |  | 124 | 132 | 83 | 80 |
| Constancy |  | 126 | 155 | 60 | 78 |
| Mystery |  | 88 | 157 | 58 | 116 |
| Self-blame |  | 41 | 193 | 22 | 163 |
| PBPI |  | 118 | 168 | 47 | 86 |
|  |  |  |  |  |  |
| Rumination |  | 136 | 147 | 68 | 68 |
| Magnification |  | 110 | 162 | 53 | 94 |
| Helplessness |  | 110 | 181 | 34 | 94 |
| PCS |  | 126 | 159 | 56 | 78 |

1. SENSITIVITY = TP/D+
2. SPECIFITY = TN/D-
3. PRECISION = TP/T+
4. ACCURACY = (TP+TN)/*n*
5. D+ = high pain group
6. D- = low pain group
7. T+ = TP + FP
